# Sex-specific impact of mild obesity on the prognosis of ST-segment elevation myocardial infarction

**DOI:** 10.1038/s41598-024-52515-4

**Published:** 2024-01-26

**Authors:** Lingling Zhang, Zhican Liu, Yunlong Zhu, Jianping Zeng, Haobo Huang, Wenbin Yang, Ke Peng, Mingxin Wu

**Affiliations:** 1 Department of Cardiology, Xiangtan Central Hospital, Xiangtan, 411100 China; 2 Graduate Collaborative Training Base of Xiangtan Central Hospital, Hengyang Medical School, University of South China, Hengyang, 421001 Hunan China; 3 Department of Cardiology, The Second Xiangya Hospital of Central South University, Changsha, 410011 Hunan China; 4 Department of Scientific Research, Xiangtan Central Hospital, Xiangtan, 411100 China; 5 Medical Department, Xiangtan Central Hospital, Xiangtan, 411100 China

**Keywords:** Cardiology, Interventional cardiology

## Abstract

This study aimed to clarify the existence of the mild obesity paradox in patients with ST-segment elevation myocardial infarction (STEMI) and assess the impact of mild obesity on the prognosis of STEMI. A retrospective cohort study was conducted on STEMI patients who underwent percutaneous coronary intervention at Xiangtan Central Hospital from January 1, 2020 to July 31, 2022. After excluding individuals with a body mass index (BMI) of no less than 35 kg/m^2^, subjects were divided into the mildly obese group (BMI, 30–35 kg/m^2^) and non-obese group (BMI < 30 kg/m^2^). The cardiovascular events and death were deemed the composite endpoints and were employed as the outcome event. The study recruited 664 patients with STEMI, including 515 males and 149 females. The mildly obese group of male patients exhibited a lower incidence of composite endpoints than the non-obese group (22.4% vs. 41.3%, *P* < 0.001). For female patients, no significant difference was observed in the incidence of composite endpoints between the two groups (43.6% vs. 43.8%, *P* = 0.987). After adjusting for confounding factors, the multivariable Cox regression analysis revealed mild obesity as an independent protective factor for male patients [hazard ratio (HR) 0.47; 95% confidence interval (CI) 0.32–0.69; *P* < 0.001]. Nevertheless, mild obesity was not associated with the prognosis of female patients (HR 0.96; 95% CI 0.47–1.94; *P* = 0.9). In male STEMI patients, mild obesity presented a paradoxical effect in improving the prognosis and functioned as an independent protective factor for the prognosis of STEMI. However, no association between mild obesity and prognosis was found in female patients, possibly due to distinct physiological and metabolic characteristics between male and female patients, which deserved further investigation and validation.

## Introduction

With the progress of society and living standards, obesity has become an increasingly critical concern. It is recognized as a substantial risk factor for cardiovascular diseases (CVD) and poses a significant challenge to global public health^1^. The World Health Organization (WHO) estimates that at least 2.6 billion adults worldwide are overweight, with around 1 billion of them being obese^2^. Obesity has recently been confirmed to be intimately linked with various CVD, such as coronary heart disease, hypertension, and diabetes^3^, indicating that it can significantly influence the onset, development, and prognosis of CVD^4^. Therefore, in-depth research on the relationship between obesity and CVD is crucial for clinical and public health.

Nevertheless, some studies have identified an unanticipated phenomenon, namely the obesity paradox^5,6^, which means that obese individuals display better prognoses than normal-weight individuals for specific CVD^7^, such as heart failure and chronic kidney disease^8^. Although the obesity paradox is controversial, an increasing number of studies indicate that obesity may not only negatively affect the prognosis of patients having some CVD^9^. ST-segment elevation myocardial infarction (STEMI) is a severe CVD that significantly affects the quality of life and survival rate of patients^10–13^. However, the role of obesity paradox in STEMI patients remains unclear^14,15^. Therefore, this research aims to examine the impact of mild obesity on the prognosis of STEMI patients to offer novel insights for clinical management and preventive measures for such cases.

## Methods

### Study design and participants

**Figure 1:**
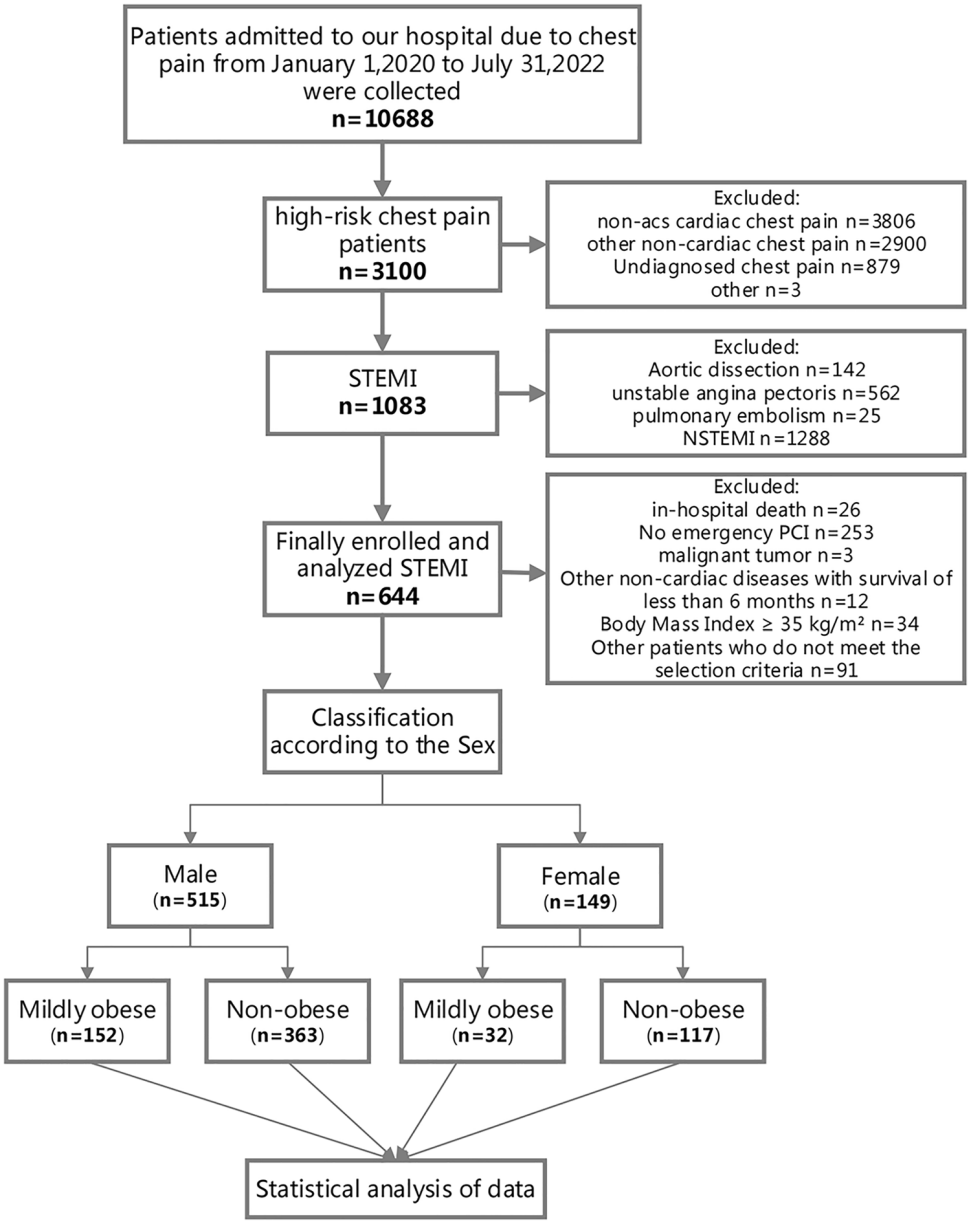
Flow diagram for participant screening, eligibility and analysis.

This retrospective cohort study enrolled 664 STEMI patients who underwent percutaneous coronary intervention (PCI) at Xiangtan Central Hospital between January 1, 2020 and July 31, 2022 (Fig. 1). The inclusion criteria were as follows: (1) Patients with first-episode STEMI based on the 2017 ESC Guidelines for the management of acute myocardial infarction^16^; and (2) patients receiving emergent PCI. The exclusion criteria included: (1) Age < 18 years; (2) lack of essential data; (3) deaths during hospitalization; (4) patients who did not undergo PCI; (5) patients with a body mass index (BMI) ≥ 35 kg/m^2^; and (6) patients with an expected survival time of fewer than 6 months due to malignant tumors or other non-cardiac diseases. Based on the WHO criteria^2^, patients were classified into the mildly obese group (BMI, 30–35 kg/m^2^) and the non-obese group (BMI < 30 kg/m^2^). We further stratified the study population by sex.

### Data collection and variable definitions

The patient’s records were retrieved from the hospital’s electronic medical record system and the national chest pain platform. These records comprised demographic information, past history, biochemical indicators at admission, medication usage, and PCI treatment-related details. During the specified study period, all eligible patients with STEMI and mild obesity in our center were consecutively included to ensure a systematic and fair selection of participants.

### Follow-up and outcome measures

We followed up with study participants until January 31, 2023. A specialized team consisting of five experienced cardiovascular physicians and two nurses collected information on outcome events through outpatient, telephone follow-up, and community registration. The primary composite endpoint was death and several cardiovascular events, including non-fatal myocardial infarction, ischemic stroke, and readmission due to angina, heart failure, bleeding, and revascularization.

### Ethics and informed consent

This study was approved by the Ethics Committee of Xiangtan Central Hospital (Xiangtan, China) (Ethics Approval No. 2023-02-001) and adhered to the Declaration of Helsinki. The requirement for individual informed consent was waived because the study was retrospective in nature and simply collected anonymous data without intervening in the patient’s treatment.

### Statistical analysis

The continuous variables were compared with the t-test or Mann–Whitney U test. The categorical variables were compared using the chi-square test. Kaplan–Meier survival curves were estimated and compared with the log-rank test. A Cox proportional hazards model was used for multivariable analysis to ascertain the independent effect of mild obesity on the prognosis of STEMI. Results were presented as the hazard ratio (HR) and 95% confidence interval (CI). P-values were obtained using the Kruskal–Wallis rank sum test or Fisher exact probability test. Results were considered significant when a *P* value < 0.05. Statistical analyses were performed using R version 4.2.0 (http://www.R-project.org) and EmpowerStats software (www.empowerstats.com, X&Y Solutions, Inc. Boston, MA).

## Results

### Baseline characteristics

This study enrolled 664 STEMI patients, including 515 males and 149 females. In the male patient cohort, the mildly obese group exhibited a younger mean age (57.3 ± 11.3 vs. 61.9 ± 12.3 years, *P* < 0.001) and a lower prevalence of renal dysfunction (9.2% vs. 17.1%, *P* = 0.022), atrial fibrillation (3.3% vs. 9.9%, *P* = 0.011), and chronic obstructive pulmonary disease (COPD) (9.2% vs. 17.6%, *P* = 0.015) than the non-obese group. Nevertheless, the mildly obese group showed a higher incidence of hyperlipidemia (48.7% vs. 35.8%, *P* = 0.006). In the female patient cohort, the mildly obese group displayed a younger mean age (64.6 ± 10.3 vs. 69.7 ± 9.0 years, *P* = 0.006) and a higher incidence of hyperlipidemia (50.0% vs. 28.2%, *P* = 0.02) than the non-obese group. Significant differences in the composite endpoints between the two groups were observed among male patients (22.4% vs. 41.3%, *P* < 0.001) but not among female patients (43.8% vs. 43.6%, *P* = 0.987) (Table 1).

**Table 1:** Baseline characteristics of mild obesity stratification after sex grouping.

	Male			Female		
	Obese (n = 152)	Non-obese (n = 363)	*P*-value	Obese (n = 32)	Non-obese (n = 117)	*P*-value
Demographics						
Age, years	57.3 ± 11.3	61.9 ± 12.3	** < 0.001**	64.6 ± 10.3	69.7 ± 9.0	**0.006**
Age ≥ 70, N (%)	21 (13.8%)	102 (28.1%)	** < 0.001**	10 (31.2%)	64 (54.7%)	**0.019**
Body mass index, kg/m^2^	30.2 ± 0.7	23.6 ± 2.3	** < 0.001**	30.4 ± 1.0	23.7 ± 2.2	** < 0.001**
Cardiac risk factors and co-morbidities, N (%)						
Smoker	108 (71.1%)	255 (70.2%)	0.855	108 (71.1%)	255 (70.2%)	0.606
History of alcohol intake	23 (15.1%)	67 (18.5%)	0.365	1 (3.1%)	4 (3.4%)	0.935
Hyperlipidemia	74 (48.7%)	130 (35.8%)	**0.006**	16 (50.0%)	33 (28.2%)	**0.020**
Hypertension	82 (53.9%)	202 (55.6%)	0.723	22 (68.8%)	76 (65.0%)	0.689
Atrial fibrillation	5 (3.3%)	36 (9.9%)	**0.011**	0 (0.0%)	11 (9.4%)	0.071
Diabetes mellitus	44 (28.9%)	92 (25.3%)	0.398	13 (40.6%)	37 (31.6%)	0.339
Hyperthyroidism	5 (3.3%)	6 (1.7%)	0.241	1 (3.1%)	3 (2.6%)	0.862
Stroke	14 (9.2%)	47 (12.9%)	0.231	8 (25.0%)	17 (14.5%)	0.160
Valvular heart disease	16 (10.5%)	58 (16.0%)	0.108	4 (12.5%)	25 (21.4%)	0.262
Cardiomyopathy	5 (3.3%)	12 (3.3%)	0.992	1 (3.1%)	8 (6.8%)	0.435
Chronic obstructive pulmonary disease	14 (9.2%)	64 (17.6%)	**0.015**	1 (3.1%)	8 (6.8%)	0.435
Renal insufficiency	14 (9.2%)	62 (17.1%)	**0.022**	3 (9.4%)	19 (16.2%)	0.332
Killip classification ≥ 2	50 (32.9%)	145 (39.9%)	0.132	13 (40.6%)	54 (46.2%)	0.577
Treatment, N (%)						
Beta-blocker	140 (92.1%)	314 (86.5%)	0.073	30 (93.8%)	106 (90.6%)	0.576
Spironolactone	38 (25.0%)	84 (23.1%)	0.651	3 (9.4%)	27 (23.1%)	0.087
Angiotensin-converting enzyme inhibitors	48 (31.6%)	122 (33.6%)	0.655	14 (43.8%)	41 (35.0%)	0.366
Angiotensin receptor blockers	48 (31.6%)	129 (35.5%)	0.388	14 (43.8%)	32 (27.4%)	0.075
Statins	151 (99.3%)	360 (99.2%)	0.842	32 (100.0%)	115 (98.3%)	0.457
Antiplatelet drugs	151 (99.3%)	360 (99.2%)	0.842	32 (100.0%)	116 (99.1%)	0.600
Clinical outcomes, N (%)						
Composite endpoint	34 (22.4%)	150 (41.3%)	** < 0.001**	14 (43.8%)	51 (43.6%)	0.987

The population was classified according by obesity stratification after sex grouping. Values for continuous variables are given as means ± SD.

Bold represent significant values (P < 0.05).

### Clinical outcomes

**Figure 2:**
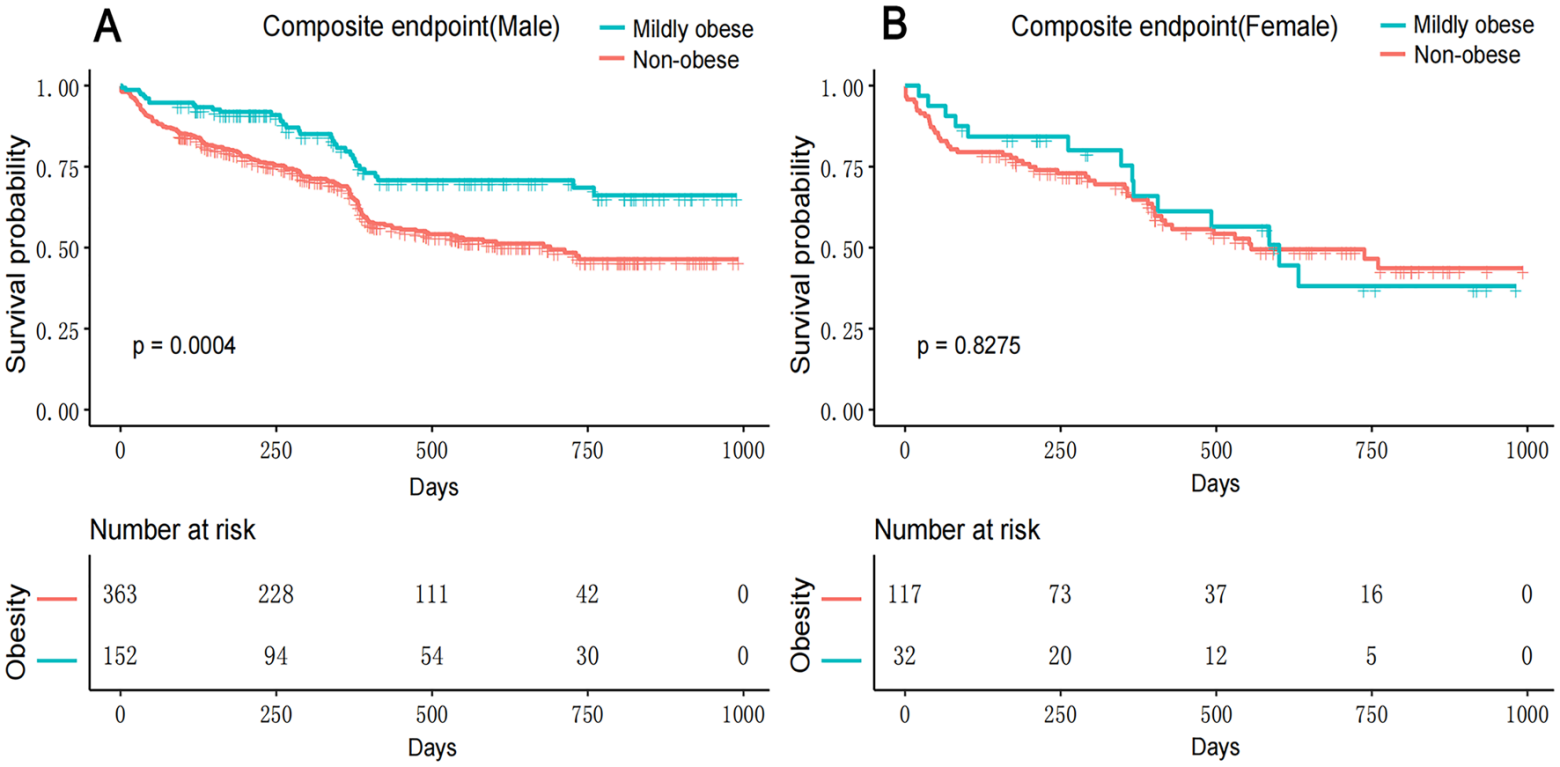
Trend plot of the composite endpoint for the mildly obese and non-obese groups. (**A**) Cumulative incidence of the composite endpoint in male. (**B**) Cumulative incidence of the composite endpoint in female.

The unadjusted analysis (Table 2, Model I) showed that the risk ratio of mildly obese patients to non-obese patients was 0.51 for the male cohort (95% CI 0.35–0.74; *P* = 0.0004) (Fig. 2A), 0.94 for the female cohort (95% CI 0.52–1.69; *P* = 0.8275) (Fig. 2B), and 0.59 for the overall population (95% CI 0.43–0.81; *P* = 0.0011).

**Table 2:** Impact of mild obesity on clinical outcomes.

	Male		Female		Total	
	HR (95% CI)	*P*-value	HR (95% CI)	*P*-value	HR (95% CI)	*P*-value
Model I						
Non-obese	Reference					
Mild obese	0.51 (0.35, 0.74)	**0.0004**	0.94 (0.52, 1.69)	0.8275	0.59 (0.43, 0.81)	**0.0011**
Model II						
Non-obese	Reference					
Mild obese	0.53 (0.36, 0.77)	**0.0008**	0.97 (0.53, 1.79)	0.9277	0.62 (0.45, 0.85)	**0.003**
Model III						
Non-obese	Reference					
Mild obese	0.51 (0.35, 0.74)	**0.0005**	1.02 (0.51, 2.03)	0.9585	0.61 (0.44, 0.85)	**0.0031**
Model IV						
Non-obese	Reference					
Mild obese	0.47 (0.32, 0.69)	**0.0001**	0.96 (0.47, 1.94)	0.9003	0.59 (0.43, 0.82)	**0.0017**

Hazard ratios from Cox proportional hazards regressions.

Model I adjust for: None.

Model II adjust for: Age.

Model III adjust for: Age; Smoker; Drinker; Hyperlipidemia; Hypertension; Atrial fibrillation; Diabetes mellitus; Hyperthyroidism; Stroke; Valvular heart disease; Cardiomyopathy; Chronic obstructive pulmonary disease; Renal insufficiency; Killip classification.

Model IV adjust for: Age; Smoker; Drinker; Hyperlipidemia; Hypertension; Atrial fibrillation; Diabetes mellitus; Hyperthyroidism; Stroke; Valvular heart disease; Cardiomyopathy; Chronic obstructive pulmonary disease; Renal insufficiency; Killip classification; Beta-blocker; Spironolactone; Angiotensin-converting enzyme inhibitors; Angiotensin receptor blockers; Statins; Antiplatelet Drugs.

*HR* hazard ratio, *CI* confidence interval.

Bold represent significant values (P < 0.05).

After adjusting for age (Table 2, Model II), the risk ratio of mildly obese patients to non-obese patients was 0.53 for the male cohort (95% CI 0.36–0.77; *P* = 0.0008), 0.97 for the female cohort (95% CI 0.53–1.79; *P* = 0.9277), and 0.62 for the overall population (95% CI 0.45–0.85; *P* = 0.0030).

After adjusting for demographic characteristics, cardiac risk factors, and comorbidities (Table 2, Model III), the risk ratio of mildly obese patients to non-obese patients was 0.51 for the male cohort (95% CI 0.35–0.74; *P* = 0.0005), 1.02 for the female cohort (95% CI 0.51–2.03; *P* = 0.9585), and 0.61 for the overall population (95% CI 0.44–0.85; *P* = 0.0031).

After adjusting for demographic characteristics, cardiac risk factors, comorbidities, and treatment strategies (Table 2, Model IV), the risk ratio of mildly obese patients to non-obese patients was 0.47 for the male cohort (95% CI 0.32–0.69; *P* = 0.0001), 0.96 for the female cohort (95% CI 0.47–1.94; *P* = 0.9003), and 0.59 for the overall population (95% CI 0.43–0.82; *P* = 0.0017).

### Independent risk factors associated with outcome events

We initially conducted univariate and multivariate analyses to investigate risk factors associated with outcome events. The findings are summarized below (Table 3).

**Table 3:** Cox proportional hazards regression model analysis for risk of composite endpoint.

	Univariable HR (95% CI)	Wald	P value	Multivariable HR (95% CI)	P value
Antiplatelet drugs	0.04 (0.01, 0.09)	**50.02**	** < 0.0001**	0.03 (0.01, 0.10)	** < 0.0001**
Killip classification ≥ 2	1.66 (1.29, 2.13)	**15.90**	** < 0.0001**	1.53 (1.18, 1.98)	**0.0013**
Mild obesity	0.58 (0.43, 0.80)	**11.25**	**0.0008**	0.61 (0.44, 0.84)	**0.0027**
Cardiomyopathy	2.03 (1.28, 3.20)	**9.16**	**0.0025**	1.78 (1.12, 2.84)	**0.0150**
Age ≥ 70	1.48 (1.14, 1.91)	**8.87**	**0.0029**	1.22 (0.93, 1.60)	0.1580
Statins	0.23 (0.09, 0.63)	**8.33**	**0.0039**	0.92 (0.23, 3.64)	0.9059
Valvular heart disease	1.57 (1.15, 2.13)	**8.21**	**0.0042**	1.34 (0.97, 1.85)	0.0789
Stroke	1.50 (1.07, 2.10)	**5.45**	**0.0196**	1.29 (0.91, 1.84)	0.1493
Atrial fibrillation	1.62 (1.08, 2.43)	**5.35**	**0.0207**	1.18 (0.77, 1.80)	0.4440
Hypertension	1.30 (1.01, 1.68)	**4.08**	**0.0434**	1.28 (0.99, 1.66)	0.0645
Spironolactone	1.30 (0.98, 1.74)	3.24	0.0719		
Beta-blocker	0.75 (0.52, 1.09)	2.31	0.1289		
Diabetes mellitus	1.23 (0.94, 1.61)	2.20	0.1381		
Renal insufficiency	1.28 (0.92, 1.76)	2.18	0.1402		
Male	0.81 (0.61, 1.08)	2.08	0.1490		
Angiotensin receptor blockers	0.86 (0.66, 1.11)	1.34	0.2473		
Smoker	0.87 (0.68, 1.12)	1.17	0.2798		
Hyperlipidemia	0.88 (0.68, 1.14)	0.99	0.3203		
History of alcohol intake	0.84 (0.58, 1.22)	0.86	0.3541		
Chronic obstructive pulmonary disease	0.84 (0.57, 1.24)	0.75	0.3850		
Hyperthyroidism	0.65 (0.24, 1.75)	0.73	0.3936		
Angiotensin-converting enzyme inhibitors	0.97 (0.75, 1.26)	0.06	0.8130		

Hazard ratios from Cox proportional hazards regressions.

*HR* hazard ratio, *CI* confidence interval;

Bold represent significant values (P < 0.05).

#### Antiplatelet agents

The univariate analysis showed that administering antiplatelet agents significantly reduced the risk of outcome events (HR 0.04; 95% CI 0.01–0.09; *P* < 0.0001). The multivariate analysis confirmed that it remained an independent protective factor (HR 0.03; 95% CI 0.01–0.10; *P* < 0.0001).

#### Killip classification

The univariate analysis indicated a significant relationship between Killip classification and an elevated risk of outcome events (HR 1.66; 95% CI 1.29–2.13; *P* < 0.0001). The multivariate analysis showed that Killip classification was an independent risk factor (HR 1.53; 95% CI 1.18–1.98; *P* = 0.0013).

#### Mild obesity

The univariate analysis revealed that mild obesity could significantly decrease the risk of outcome events (HR 0.58; 95% CI 0.43–0.80; *P*, 0.0008). In the multivariate analysis, it remained an independent protective factor (HR 0.61; 95% CI 0.44–0.84; *P* = 0.0027).

#### Cardiomyopathy

The univariate analysis demonstrated a significant relationship between cardiomyopathy and an elevated risk of outcome events (HR 2.03; 95% CI 1.28–3.20; *P* = 0.0025). The multivariate analysis further established it as an independent risk factor (HR 1.78; 95% CI 1.12–2.84; *P* = 0.015).

Other factors, such as age, valvular heart disease, stroke, atrial fibrillation, and hypertension, emerged as risk factors for outcome events in the univariate analysis. However, their impacts were not statistically significant in the multivariate analysis. Besides, lipid-lowering drugs, ARBs, smoking, hyperlipidemia, alcohol consumption, COPD, hyperthyroidism, and ACEIs exhibited no significant association with outcome events in the univariate and multivariate analyses.

### Stratified analysis

**Figure 3:**
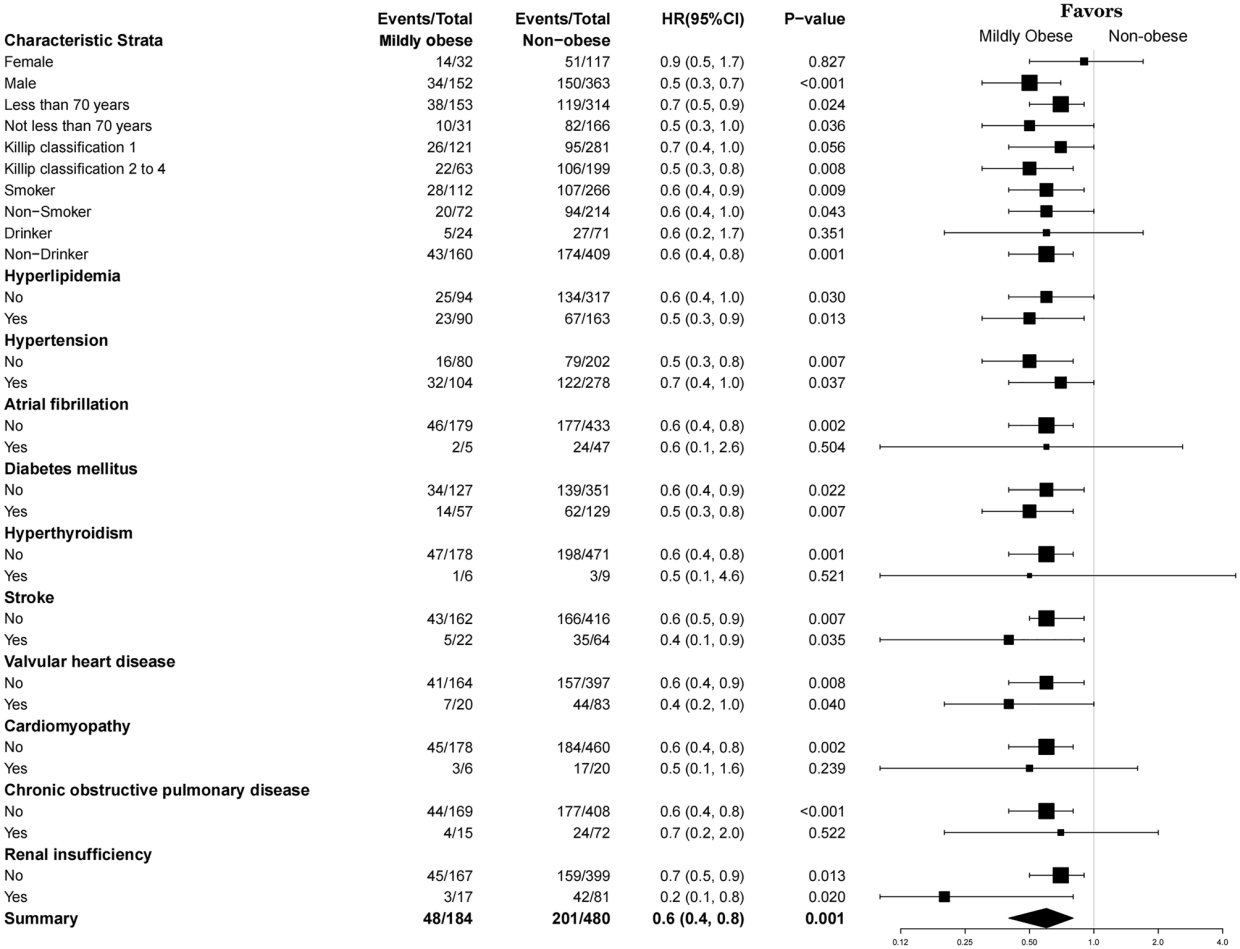
Forest plot: Relationship between mild obesity and composite endpoint events, stratified by multiple dichotomous variables based on obesity grouping.

We conducted a comprehensive stratified analysis for multiple binary variables. Forest plots (Fig. 3) illustrated the relationship between mild obesity (independent variable) and composite endpoint events (dependent variable). The stratified analysis showed that mild obesity exerted a protective effect on the patient’s prognosis in most subgroups, particularly under the following conditions: age ≥ 70 or < 70 years, smoker or non-smoker, non-drinker, with or without hyperlipidemia, with or without hypertension, without atrial fibrillation, with or without diabetes, without hyperthyroidism, with or without stroke, with or without valvular heart disease, without cardiomyopathy, without COPD, with or without renal insufficiency, and Killip classification ≥ 2 (all *P* < 0.05). In contrast, the association between mild obesity and composite endpoint events did not achieve statistical significance in subgroups of females, alcohol consumers, patients with atrial fibrillation, hyperthyroidism, cardiomyopathy, COPD, and Killip class I (all *P* values > 0.05).

We carefully considered and analyzed numerous significant factors that might affect the prognosis of STEMI patients. To provide a comprehensive overview, we compared other important prognostic indicators between the mildly obese and non-obese groups (Supplementary Table 1).

### Ethics approval and consent to participate

The study protocol was approved by the Ethics Committee of Xiangtan Central Hospital (Xiangtan, China, No. 2023-02-001) and conformed to the principles outlined in the Declaration of Helsinki. The need for informed consent was waived by the ethics committee Review Board of Xiangtan Central Hospital, because of the retrospective nature of the study.

## Discussion

The study revealed that mild obesity functioned as an independent protective factor for the composite endpoint in male STEMI patients after adjusting for confounding variables. However, no association was identified between mild obesity and composite endpoint among female STEMI patients. Additionally, the administration of antiplatelet agents emerged as an independent protective factor for the incidence of the composite endpoint. In contrast, a Killip class ≥ 2 and a history of cardiomyopathy were established as independent risk factors.

The fundamental similarity between this study and prior research validated the “obesity paradox” in obese patients, suggesting that obesity was correlated with a more favorable prognosis in particular CVD. Numerous investigations have demonstrated obesity as a significant risk factor for CVD, while obese patients exhibited a better prognosis than non-obese patients under specific circumstances, such as acute myocardial infarction and heart failure^1,5,14,17,18^. This phenomenon was known as the “obesity paradox”.

The uniqueness of this study was that we further examined the influence of sex on the mild obesity paradox and revealed differences in the association of mild obesity with the prognosis of STEMI between male and female patients. Previous research has primarily concentrated on the mild obesity paradox, with less attention paid to the role of the sex factor^19,20^. In research focused on gender disparities in Acute Coronary Syndrome (ACS), there has been no observed trend supporting the obesity paradox^21^. Our investigation discovered a protective effect of mild obesity on male STEMI patients; however, this effect was not observed in female patients. This finding emphasized the importance of gender difference when assessing the impact of mild obesity on the prognosis of CVD and provided an innovative perspective for developing future interventions to address these differences.

This study suggested that the protective effect of mild obesity on the prognosis of male STEMI patients might be attributed to the following factors: (1) Obese patients were younger at onset and possessed a lower risk of CVD^6,22^; (2) obese patients demonstrated excellent myocardial reserve function and resistance to myocardial ischemia^23^; and (3) inflammatory factors and metabolic hormones in obese patients could have a protective effect on the myocardium^24^. In contrast, this protective effect was not present in female patients, possibly due to differences in the physiology, metabolism, diagnosis and treatment of coronary artery disease, characteristics of acute myocardial infarction, and coronary microvascular function associated with diabetes between male and female patients^25–29^.

### Study limitations

The main limitations of this study included the following aspects: (1) The retrospective design of the study might lead to the bias of final results; (2) the sample size was relatively small, with a particularly limited number of females and patients with BMI ≥ 35 kg/m^2^; (3) other metabolic indicators and hormone levels were not considered; (4) The limited racial diversity of the study cohort might affect the generalizability of our findings in other populations. To address these issues, future research should adopt a prospective design, increase the sample size, particularly the number of female patients, and further evaluate the impact of other metabolic indicators and hormone levels.

### Clinical implications

To tackle the above limitations, we suggest future research directions as follows: (1) Conduct prospective studies to minimize potential omissions and biases; (2) expand the research scope by increasing the sample size of female patients for a more comprehensive exploration of the impact of gender on the obesity paradox and incorporating patients with BMI ≥ 35 kg/m^2^ to determine the cut-off value of BMI associated with the obesity paradox; (3) investigate the biological mechanisms of the impact of mild obesity on the prognosis of STEMI by taking into account factors like inflammation, metabolic hormones, and hormone levels; (4) Examine other interventions, including lifestyle change and medication treatments, to improve the prognosis of obese patients with CVD.

## Conclusion

In conclusion, our study demonstrates that mild obesity is an independent protective factor for clinical outcomes in male patients with STEMI. Conversely, this protective effect was not observed in female patients. Further research is warranted to elucidate the underlying mechanism of our finding and develop potential sex-specific interventions to improve the prognosis of STEMI patients.

### Supplementary Information

Supplementary Information.

Supplementary Table 1.

## Data Availability

The datasets generated and analyzed during the current study are not publicly available due the database owner is reluctant to make them public but are available from the corresponding author upon reasonable request. If anyone wishes to request the data pertaining to this study, please contact the corresponding author, Mingxin Wu.
