# Supplementary material for: Sex-specific impact of mild obesity on the prognosis of ST-segment elevation myocardial infarction
Source: Sci Rep. 2024 Jan 26;14:2228. doi: 10.1038/s41598-024-52515-4 (PMC10817908; doi:10.1038/s41598-024-52515-4)
Supplement: Supplementary file 2 — Supplementary Table 1. [file 41598_2024_52515_MOESM2_ESM.docx]

| Supplementary Table 1 Other potentially prognostically significant indicators were compared between the mild obese and non-obese groups. | | | |
| --- | --- | --- | --- |
|  | Non-obese (n=480) | mild obese (n=184) | *P-value* |
| FMC-to-ECG | 4.44 ± 3.54 | 4.12 ± 3.42 | 0.308 |
| FMC-to-loading dose DAPT | 35.02 ± 31.75 | 34.21 ± 32.63 | 0.77 |
| diagnosis-to-loading dose DAPT | 9.56 ± 8.73 | 9.20 ± 9.49 | 0.643 |
| diagnosis-to-the first intravenous heparin | 11.35 ± 4.54 | 10.76 ± 3.69 | 0.12 |
| Troponin report time | 17.60 ± 2.31 | 17.51 ± 2.62 | 0.654 |
| consultation time（notice to arrival） | 3.37 ± 1.80 | 3.26 ± 1.69 | 0.49 |
| D-to-B time | 70.10 ± 27.66 | 68.13 ± 24.82 | 0.398 |
| total ischemic time (onset-to-reperfusion) | 438.00 ± 558.53 | 372.35 ± 402.83 | 0.146 |
| SO-to-FMC | 295.29 ± 526.55 | 246.28 ± 381.30 | 0.25 |
| FMC inhospital-to-start reperfusion | 23.66 ± 18.44 | 23.67 ± 20.67 | 0.993 |
| diagnosis and treatment time in ED | 29.83 ± 21.01 | 29.74 ± 21.19 | 0.961 |
| FMC inhospital-to-notice consultation | 11.17 ± 14.83 | 11.08 ± 10.63 | 0.94 |
| Leave ED to arrive CL | 18.66 ± 24.44 | 20.72 ± 27.58 | 0.349 |
| PCI informed consent time | 11.40 ± 10.82 | 11.32 ± 10.09 | 0.927 |
| CL activation time | 14.10 ± 11.05 | 13.03 ± 9.27 | 0.243 |
| Selection of Vessel for Puncture |  |  | 0.799 |
| Radial Artery | 446 (92.92%) | 172 (93.48%) |  |
| Femoral Artery | 34 (7.08%) | 12 (6.52%) |  |
| Major diseased vessels |  |  | 0.465 |
| Left Anterior Descending | 229 (47.71%) | 82 (44.57%) |  |
| Left Circumflex | 42 (8.75%) | 23 (12.50%) |  |
| Right coronary artery | 204 (42.50%) | 78 (42.39%) |  |
| Left main coronary artery | 5 (1.04%) | 1 (0.54%) |  |
| Degree of vessel narrowing |  |  | 0.687 |
| 90~99% | 138 (28.75%) | 50 (27.17%) |  |
| 100% | 342 (71.25%) | 134 (72.83%) |  |
| Number of stents implanted |  |  | 0.206 |
| 0 | 76 (15.83%) | 21 (11.41%) |  |
| 1 | 319 (66.46%) | 135 (73.37%) |  |
| 2 | 77 (16.04%) | 23 (12.50%) |  |
| 3 | 8 (1.67%) | 5 (2.72%) |  |
| Complications |  |  | 0.309 |
| none | 334 (69.58%) | 146 (79.35%) |  |
| shock | 34 (7.08%) | 6 (3.26%) |  |
| mechanical complications | 5 (1.04%) | 1 (0.54%) |  |
| stroke | 4 (0.83%) | 1 (0.54%) |  |
| bleeding | 9 (1.88%) | 4 (2.17%) |  |
| infection | 79 (16.46%) | 24 (13.04%) |  |
| coronary artery thrombosis formation | 1 (0.21%) | 1 (0.54%) |  |
| respiratory failure | 3 (0.62%) | 0 (0.00%) |  |
| recurrent myocardial infarction | 2 (0.42%) | 0 (0.00%) |  |
| in-hospital death | 9 (1.88%) | 1 (0.54%) |  |
| Abbreviations:CI:conﬁdence interval; FMC: first medical contact; ECG:electrocardiogram; DAPT:dual antiplatelet therapy; D-to-B:door-to-balloon ; SO:symptom onset; ED:emergency department; CL: catheter lab; PCI:percutaneous transluminal coronary intervention; | | | |
